# Resveratrol as a BCL6 natural inhibitor suppresses germinal center derived Non-Hodgkin lymphoma cells growth

**DOI:** 10.1007/s11418-024-01873-4

**Published:** 2025-01-15

**Authors:** Yajing Xing, Chunbin Tan, Zhoujiang Liu, Yanqi Liu, Simei Liu, Guixue Wang, Yadong Zhong

**Affiliations:** 1 Chongqing Academy of Chinese Materia Medica, Chongqing University of Chinese Medicine, Chongqing, 402760 China; 2 College of Traditional Chinese Medicine, Chongqing University of Chinese Medicine, Chongqing, 402760 China; 3 Key Laboratory for Biorheological Science and Technology of Ministry of Education, State and Local Joint Engineering Laboratory for Vascular Implants, Bioengineering College of Chongqing University, Chongqing, 400030 China; 4 College of Public Health, Chongqing Medical University, Chongqing, 401331 China

**Keywords:** NHL, BCL6, BTB domain, Natural inhibitor, GC

## Abstract

Non-Hodgkin lymphomas (NHL), including diffuse large B-cell lymphoma (DLBCL), Burkitt lymphoma (BL), and follicular lymphoma (FL), predominantly arise from B cells undergoing germinal center (GC) reactions. The transcriptional repressor B-cell lymphoma 6 (BCL6) is indispensable for GC formation and contributes to lymphomagenesis via its BTB domain-mediated suppression of target genes. Dysregulation of BCL6 underpins the pathogenesis of GC-derived NHL. While pharmacological targeting the BCL6-BTB domain has shown therapeutic promise, natural product-based inhibitors remain underexplored. In this study, resveratrol, a polyphenolic compound derived from grapes, was identified as a potent BCL6 inhibitor through a comprehensive screen of traditional Chinese medicine monomers using Homogeneous Time-Resolved Fluorescence (HTRF) assay. As a BCL6 natural inhibitor, resveratrol effectively disrupted the BCL6/SMRT interaction, reactivated suppressed gene expression, and inhibited the proliferation of GC-derived NHL cells. It also exhibited synergistic efficacy when combined with EZH2 and PRMT5 inhibitors. In vivo, resveratrol suppressed GC formation, reduced follicular helper T-cell frequencies, impaired class-switch recombination, and disrupted immunoglobulin affinity maturation. Furthermore, it markedly inhibited the progression of GC-derived NHL in animal models. Our findings demonstrate that resveratrol functions as a natural BCL6 inhibitor with significant therapeutic potential for the treatment of GC-derived NHL.

**Graphical abstract:**

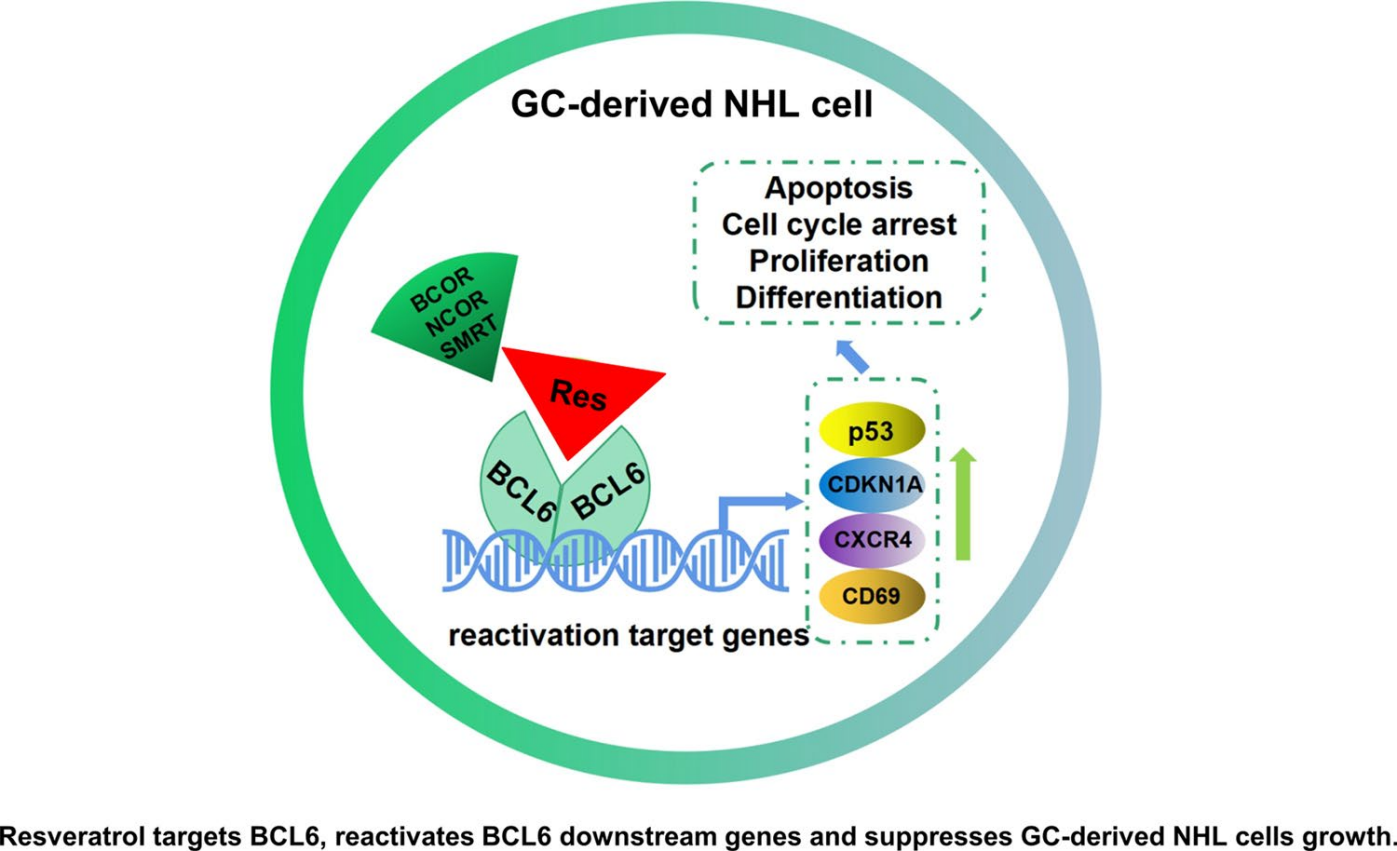

**Supplementary Information:**

The online version contains supplementary material available at 10.1007/s11418-024-01873-4.

## Introduction

Non-Hodgkin lymphomas (NHL) are among the most prevalent malignancies worldwide, comprising a diverse spectrum of lymphoid neoplasms with distinct biological behaviors and clinical outcomes [1–4]. Approximately 70–80% of NHL cases are derived from the germinal center (GC) reaction, encompassing diffuse large B-cell lymphoma (DLBCL), follicular lymphoma (FL), and Burkitt lymphoma (BL) [5, 6]. DLBCL, the most common NHL subtype, is highly aggressive and associated with substantial disease burden [7]. In contrast, FL, an indolent lymphoma, is characterized by prolonged survival but frequent relapses and a risk of transformation into aggressive DLBCL [8, 9]. BL, although rare and primarily affecting younger populations, demands intensive therapeutic approaches due to its high proliferative index [5, 10]. The standard treatment for B-cell NHL integrates rituximab with chemotherapy, yielding favorable initial responses for many patients. Nevertheless, treatment resistance and relapse are frequent, necessitating alternative therapeutic strategies, including autologous stem cell transplantation, which faces limitations in efficacy and accessibility [6, 11]. CAR-T cell therapy has become an emerging strategy for B-NHL therapy but remains constrained by high costs and limited availability in many regions [7]. These challenges underscore the critical need for innovative, scalable, and effective therapies tailored to GC-derived NHL.

Studies have shown that the progression of GC-derived NHL are closely related to the abnormal expression of the B cell lymphoma 6 (BCL6) protein [5, 12–14]. BCL6, a pivotal transcriptional repressor, orchestrates the formation and function of GCs, transient structures in secondary lymphoid organs where B cells undergo clonal expansion, class-switch recombination, and somatic hypermutation in response to T cell-dependent antigens [15]. These processes are essential for generating high-affinity memory B cells and plasma cells, integral to humoral immunity [16, 17]. Chromosomal translocations or point mutations that sustain BCL6 overexpression are key drivers of GC-derived lymphomagenesis [12, 18]. Notably, GC-derived NHL cells retain a dependency on BCL6 for survival and proliferation, reflecting their GC origin [5]. Consequently, targeting BCL6 has emerged as a promising therapeutic approach for treating GC-derived NHL [19, 20].

BCL6 belongs to the BTB/POZ family and consists of three domains: the N-terminal BTB/POZ domain, a central linker region, and the C-terminal zinc finger domain [21]. The BTB domain mediates BCL6 transcriptional repression by forming homodimers that recruit corepressors, including SMRT, NCOR, and BCOR, in a mutually exclusive manner [20]. Mutating two amino acids in the BTB domain of BCL6 (N21K, H116A) in mice renders BCL6 unable to exert its transcriptional repressive function, resembling the phenotype of BCL6 knockout mice [22]. Inhibiting BCL6-BTB will inhibit GC formation and lymphoma cell proliferation, without causing significant toxic side effects or macrophage-driven inflammatory responses [22, 23]. As such, targeting the BCL6 BTB domain is an effective strategy for treating GC-derived NHL.

To date, reported BCL6 inhibitors are mostly peptides and synthetic small molecules. Notable examples include peptide inhibitors such as BPI [24] and RI-BPI [25], the small molecules 79–6 [26] and FX1 [27], and the BCL6 degrader BI3802 [28]. Despite these advances, the identification of natural products targeting BCL6 remains limited. Here, we constructed an HTRF assay to screen natural product library and identified resveratrol as a BCL6 inhibitor. Resveratrol, a polyphenolic compound derived from grapes, is recognized for its antioxidant, anti-cancer, anti-inflammatory, and cardiovascular benefits [29]. Prior investigations have demonstrated that resveratrol can induce cell cycle arrest and apoptosis in FL (OCI-LY8) derived from DLBCL [30]. However, resveratrol’s activity in vivo and broader therapeutic potential across GC-derived NHL subtypes remain unexplored. This study explores the efficacy and mechanism of resveratrol in inhibiting GC-derived NHL both in vivo and in vitro. By uncovering its role as a natural BCL6 inhibitor, we highlight its potential as a novel therapeutic agent, offering a new avenue for addressing unmet clinical needs in NHL treatment.

## Material and methods

### Mammalian cell culture and animals

SUDHL4, Farage, Ramos, Raji, NCM460, LO2, HAF, and 293 T cell lines were obtained from ATCC (American Type Culture Collection, Manassas, VA, USA), while the DOHH2 cell line was procured from DSMZ (German Collection of Microorganisms and Cell Cultures, Braunschweig, Germany) and PNT1A cells were sourced from ECACC (European Collection of Authenticated Cell Cultures, Salisbury, UK). SUDHL4, Farage, Ramos, Raji, DOHH2, NCM460, PNT1A, and LO2 cells were cultured in RPMI-1640 medium (Gibco, Grand Island, NY, USA) supplemented with 10% fetal bovine serum (FBS; Invitrogen, Carlsbad, CA, USA) and 1% penicillin–streptomycin (Invitrogen, Carlsbad, CA, USA). The 293 T cells were maintained in Dulbecco's Modified Eagle Medium (DMEM, Gibco, Grand Island, NY, USA) containing 10% FBS and 1% penicillin–streptomycin, whereas HAF cells were cultured in DMEM supplemented with 10% FBS, 1% penicillin–streptomycin, and 2 mM L-glutamine (Gibco, Grand Island, NY, USA). All cell lines were maintained at 37 °C in a humidified atmosphere containing 5% CO₂. Cells were routinely screened for Mycoplasma contamination using polymerase chain reaction (PCR) and were utilized for experiments within passages 6 to 10 to ensure consistency and viability.

Animal experiments were conducted using models obtained from the Animal Center of Chongqing Academy of Chinese Materia Medica. All animal procedures were carried out in compliance with the ethical guidelines of the Chongqing Academy of Chinese Materia Medica and were approved by its Animal Investigation Committee (DWS2023-03).

### Protein and SMRT peptide

Cloning of the DNA sequence encoding the BCL6 BTB domain (residues 5–129) was cloned into a pGEX vector to incorporate a GST tag, facilitating expression in E. coli BL21 (DE3). The mutants R24A, G55A, R28A, N21A, Y58A, L25A of the BCL6 BTB produced by site-directed mutagenesis were also cloned into the same vector. The constructs were expressed and incubated for 18 h with 0.5 mM IPTG. The BCL6-BTB and mutant proteins were purified using glutathione agarose resin and dialyzed. The peptide SMRT (residues 1414–1430) featuring a His tag was from Abace Biology Company.

### HTRF assay

The assay was conducted in 384-well plates (Greiner Bio-One, 784,045), where BCL6-GST and SMRT-6His proteins were added alongside the test compound. Following 1 h incubation in dilution buffer, anti-6His-XL665 and anti-GST-Tb (Cisbio) were introduced, and the mixture was incubated overnight. The fluorescence signals were recorded at 665 nm and 620 nm using a microplate reader (BioTek Cytation5). The natural product library was sourced from Targetmol (L6700), and resveratrol (purity 99.9%) was purchased from Targetmol (T1558, Boston, MA).

### Luciferase reporter assay

The luciferase reporter construct (GAL_4_)_5_-TK-LUC and the GAL_4_-DBD-BCL6^BTB^ expression plasmid were generously provided by Dr. Ari Melnick (Department of Haematology/Oncology, Weill Cornell Medical College, New York, NY, USA). A TK-Renilla luciferase plasmid (Promega, WA, USA) was used as an internal normalization control. Co-transfections were performed in 293 T cells using Lipofectamine 2000 (Thermo Fisher Scientific) to introduce the GAL_4_-DBD-BCL6^BTB^ or GAL4-DBD expression plasmids along with the (GAL_4_)_5_-TK-LUC reporter and TK-Renilla plasmids. After 6 h transfection period, the cells were treated with compounds for 24 h. Luciferase activity was subsequently measured using the Dual-Luciferase Reporter Assay System (Promega, WA, USA).

### Surface plasmon resonance (SPR)

The BTB protein and its mutants were covalently attached to the CM5 chip (GE Healthcare) via amine coupling at a flow rate of 10 μL/min, using a 10 mM sodium acetate buffer solution. Subsequently, proteins were passed over the chip for a duration of 420 s, followed by surface deactivation with ethanolamine. The subsequent kinetic and affinity analyses were conducted in a buffer comprising 25 mM HEPES at a controlled temperature of 25 °C. Resveratrol was introduced into the flow system at varying concentrations. The resulting data were analyzed employing the Biacore T200 Plus Evaluation Software.

### Molecular docking of BCL6 and resveratrol

The three-dimensional structure of BCL6 protein was elucidated through the implementation of the AlphaFold3 algorithm. The predicted BCL6 structure was subjected to preparatory processing using UCSF Chimera software, during which hydrogen addition, charge assignment, and conformational refinement were performed. Concurrently, the three-dimensional structure of resveratrol was generated utilizing MarvinSketch and was optimized with the MMFF94 force field. The molecular docking procedure was executed via the AutoDock Tools 1.5.6. A search space encompassing the putative active site of BCL6 was delineated, and docking simulations were conducted using default parametric settings. To enhance the robustness of the results, 100 independent docking iterations were performed, from which the optimal conformation was selected based on binding energy and conformational clustering criteria. Visualization of both the BCL6 protein and the BCL6-resveratrol complex was accomplished in PyMOL 1.8.

### Real-Time quantitative PCR

Total RNA was isolated using TRIzol reagent (Invitrogen). Complementary DNA was synthesized using the PrimeScript™ RT reagent kit (Takara) following the guidelines. Quantitative Real-Time PCR was performed using SYBR (Takara).

### Mouse immunization

C57BL/6 mice were immunized with 100 µg of NP_18_-CGG in alum adjuvant. Two days post-immunization, mice received daily intraperitoneal injections of resveratrol at a dose of 100 mg/kg for 12 consecutive days. The mice were euthanized, and spleens and sera were harvested. GC B and Tfh cells in splenocytes were quantified using flow cytometry. Serum samples were analyzed for antigen-specific antibody levels using ELISA.

### GC analysis

Single-cell suspensions were prepared from spleens. The cells were washed and stained with a panel of fluorescently conjugated antibodies to identify GC B cells and Tfh cells. Antibodies included PerCP/Cy5.5-B220 (BioLegend, 103,236), FITC-FAS (BD Pharmingen, 554,257), eFluor 660-GL7 (Invitrogen, 50–5902-82), PerCP/Cy5.5 -CD4 (BioLegend, 100,434), PE-CXCR5 (BioLegend, 145,504), and APC-PD-1 (BioLegend, 109,112). Samples were acquired using a FACS Calibur flow cytometer (BD Biosciences). GC B cells were defined as B220^+^GL7^+^FAS^+^ populations, and Tfh cells were identified as CD4^+^CXCR5^+^PD-1^+^ populations.

### ELISA

Plates were coated with NP_5_-BSA or NP_23_-BSA (Southern Biotech) to capture high and low affinity NP-specific antibodies. Serum were added to the plates and incubated for 2 h. After washing, HRP-conjugated goat anti-mouse IgG secondary antibodies (Southern Biotech) were applied, followed by a substrate solution (TMB, Thermo Fisher Scientific). Reactions were terminated with 2 M sulfuric acid, and OD value was measured at 450 nm.

### Cell viability and drug synergy assay

Cells were seeded in 96-well plates at a density of 2 × 10^4^ cells per well, optimized according to cell line requirements. Next, they were treated with resveratrol for 72 h. Subsequently, 20 μL of MTS reagent was added to well. OD value at 490 nm was measured by a microplate reader (Bio-Rad). For combination treatments, cells were exposed to the PRMT5 inhibitor GSK591 and the EZH2 inhibitor GSK343 (TargetMol, USA), either individually or in fixed-ratio combinations. Drug synergy was analyzed using the Chou-Talalay method, with combination index (CI) values calculated by CalcuSyn software (version 2.0, Biosoft). CI values less than 1 were interpreted as synergistic, with CI values below 0.7 indicating strong synergy.

### Xenograft tumour model

SUDHL4 xenograft models were established by subcutaneous injection of 1 × 10⁷ SUDHL4 cells into the flanks of SCID mice. Tumors were monitored using digital calipers. When tumors reached approximately 100 mm^3^, mice were randomized into groups. Resveratrol or vehicle control was administered intraperitoneally at defined doses, with treatments continued for a predetermined duration. Tumor volume and body weight were measured twice weekly to monitor treatment efficacy and systemic toxicity. Upon study termination, tumors were excised, weighed, and processed for molecular analyses.

### Statistical analysis

The results are expressed as the mean ± SD. The significance of the difference between two groups was analyzed by Student’s t-test. For animal experiments, data were analyzed by two-way ANOVA. All experiments were performed at least three times, except for the animal experiments. All statistical analyses were carried out using GraphPad Prism 5.0 software. The significant differences in the means were determined at the level of *P < 0.05, **P < 0.01 and ***P < 0.001.

## Results

### ***Resveratrol is a BCL6***^***BTB***^*** natural inhibitor***

**Fig. 1:**
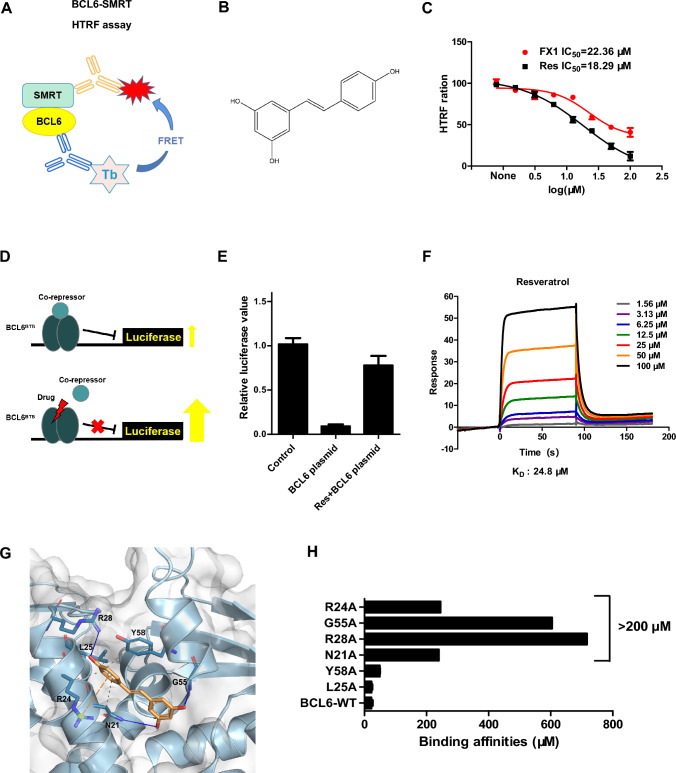
Resveratrol is identified as a BCL6^BTB^ natural inhibitor. **A** Schematic diagram of HTRF assay screening for BCL6 inhibitors. **B** Chemical structure of resveratrol. **C** Resveratrol and FX1 were tested in HTRF assay for inhibition of the BCL6-SMRT interaction. (D) Schematic diagram of dual-luciferase reporter assay. **E** Luciferase reporter assay was performed to test the activity of resveratrol against the repressor activity of BCL6^BTB^. When plasmids (GAL_4_)_5_-TK-LUC combined with GAL_4_-DBD-BCL6^BTB^, the fluorescence expression was inhibited. After adding 100 μM of resveratrol, the fluorescence was restored. **F** K_D_ of resveratrol binding to BCL6 BTB protein determined by an SPR assay. **G** Molecular docking model predicted that resveratrol bound to the BCL6-BTB domain. **H** The binding affinities of resveratrol and BCL6 mutants

BCL6 functions as an oncogene in the pathogenesis and progression of GC-derived NHL [12, 15]. To screen for natural inhibitors of BCL6, we constructed a HTRF assay to evaluate the disruption activity of BCL6^BTB^/SMRT complex formation (Fig. 1A). Through high-throughput screening of over 1,800 natural products, resveratrol emerged as a promising candidate (Fig. 1B). To confirm the reliability of our screening approach, we employed FX1, a previously reported BCL6 inhibitor, as a positive control (Supplementary Fig. 1A). Both FX1 and resveratrol were evaluated for their inhibitory effects on the BCL6-BTB/SMRT interaction. Resveratrol demonstrated an IC_50_ value of 18.29 μM, while FX1 exhibited an IC_50_ of 22.36 μM, confirming the potent inhibitory activity of resveratrol (Fig. 1C). Subsequently, we used luciferase reporter assay to assess the depression impact of resveratrol on BTB mediated transcription (Fig. 1D). In the system, the luciferase fluorescence was significantly inhibited after the introduction of BCL6. However, upon adding resveratrol at 100 μM, the fluorescence was partially restored, indicating that resveratrol can inhibit transcriptional activity of BCL6 (Fig. 1E). In addition, resveratrol exhibited stronger inhibition of BCL6 transcriptional activity compared to FX1 at 50 μM (Supplementary Fig. 1B).

To evaluate whether resveratrol directly binds to the BCL6 protein, we expressed and purified the BTB domain of BCL6 (residues 5–129). SPR analysis revealed a concentration-dependent increase in the response signal as the resveratrol concentration increased, indicating specific binding between resveratrol and the BCL6 BTB domain. The binding affinity was quantified, yielding an equilibrium dissociation constant (K_D_) of 24.8 µM (Fig. 1F). Next, we performed molecular docking simulation by using Alpha Fold 3 to elucidate the binding mode of resveratrol to BCL6. Figure 1G showed that Asn21, Leu25, and Tyr58 interacted with the hydrophobic phenolic ring of resveratrol through hydrophobic forces. Additionally, Asn21, Arg28, and Gly55 formed hydrogen bonds with the three hydroxyl groups of resveratrol. Moreover, the guanidinium cation of Arg24 established a pi-cation interaction with the hydrophobic phenolic ring of resveratrol. To experimentally validate the key amino acid residues involved in the interaction between resveratrol and BCL6, we generated a series of single-residue mutants of the predicted binding site. The mutant proteins were purified, and their binding to resveratrol was evaluated using SPR assays. As our results showed, the binding affinities between resveratrol and the BTB mutants including L25A and Y58A were only modestly changed compared with the binding affinities of resveratrol to BCL6-WT (Fig. 1H, Supplementary Fig. 2). In contrast, resveratrol did not bind several mutants including N21A, R28A, G55A, and R24A (K_D_ > 200 µM) (Fig. 1H). Thus, direct binding results demonstrated that resveratrol directly bound to the residues of N21, R28, G55, and R24. Collectively, these findings demonstrate that resveratrol is a BCL6-BTB natural inhibitor.

### *Resveratrol inhibits BCL6 transcriptional function *in vitro

**Fig. 2:**
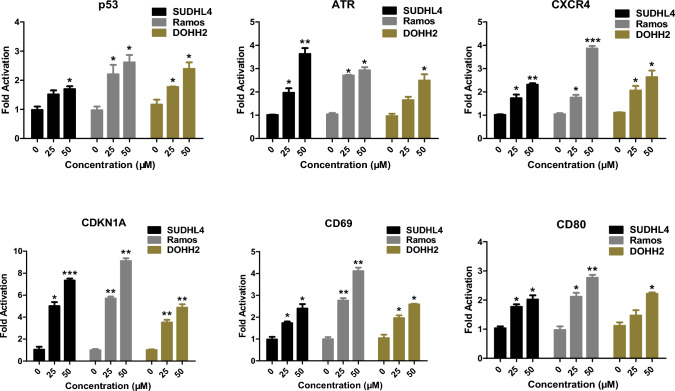
Resveratrol induces de-repression of BCL6 target genes p53, ATR, CXCR4, CDKN1A, CD69 and CD80. (*, *P* < 0.05; **, *P* < 0.01; ***, *P* < 0.001 versus control)

As an oncogene, BCL6 exerts its transcriptional repression activity by recruiting transcriptional corepressors through its BTB domain, thereby suppressing its target genes, promoting cancer cell proliferation and tumor progression [27, 31–33]. To investigate whether resveratrol can counteract this repression, we treated the DLBCL cell line SUDHL4, the BL cell line Ramos, and the FL cell line DOHH2 with resveratrol and extracted cellular RNA for qPCR analysis. As shown in Fig. 2, resveratrol was able to upregulate the expression of BCL6 downstream genes p53, ATR, CXCR4, CDKN1A, CD69, and CD80 in all three lymphoma cell lines at 25 µM. Additionally, besides being highly expressed in lymphomas, BCL6 is also highly expressed in various solid tumors such as breast cancer [34], ovarian cancer [35], and glioma [36]. Therefore, we also assessed the effects of resveratrol in breast cancer cell line (MDA-MB-231), ovarian cancer cell line (ES-2), and glioma cell line (U87). The results showed that resveratrol similarly reactivated the BCL6 target genes expression in these tumor cells (Supplementary Fig. 3). These findings indicate that resveratrol inhibits the transcriptional repression of BCL6-BTB and reactivates the expression of BCL6 downstream genes.

### *Resveratrol inhibits BCL6 biological function *in vivo

**Fig. 3:**
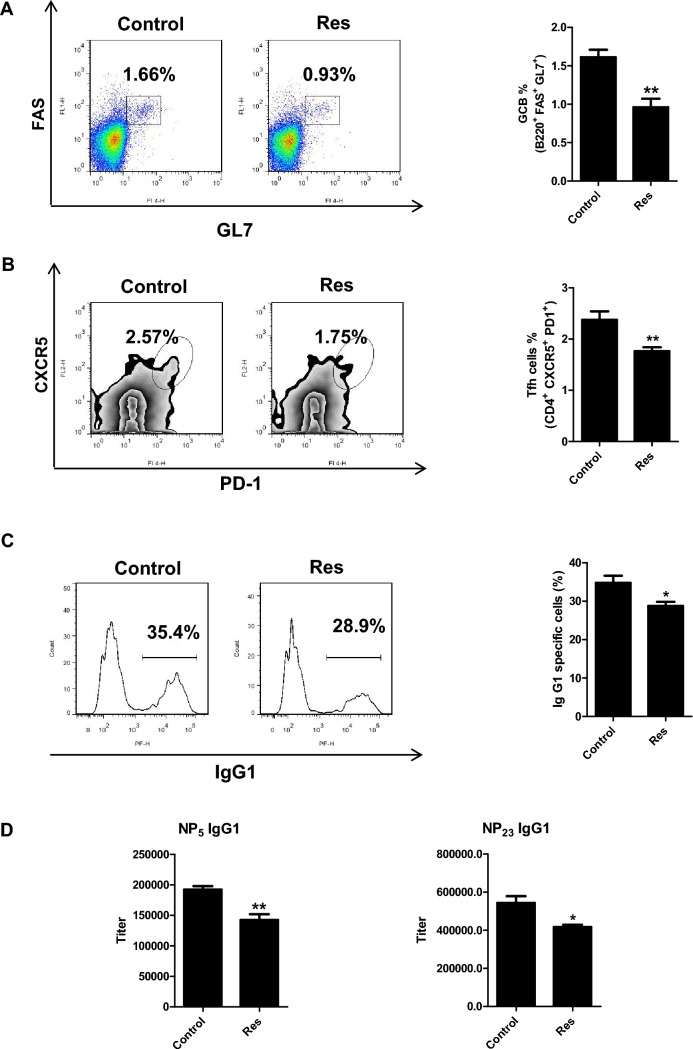
Resveratrol blocks GC formation in vivo. C57BL/6 mice were immunized with NP_18_-CGG and treated with resveratrol at a dosage of 100 mg/kg/d. **A** Flow cytometry analysis revealed that the proportion of GC B cells in the resveratrol group decreased. **B** Percentage of Tfh cells was reduced in the resveratrol group by flow cytometry. **C** Resveratrol affected class-switch recombination in flow cytometry analysis. **D** Resveratrol decreased the titers of the NP-specific immunoglobulin G1 in serum measured by ELISA with NP_5_-BSA and NP_23_-BSA. (*, *P* < 0.05; **, *P* < 0.01 versus control)

GC is a transient structure formed in peripheral lymphoid organs by mature B cells upon antigen stimulation, and the transcriptional repression function mediated by the BCL6 BTB domain is crucial for GC formation [22]. Genetic ablation of BCL6 or mutations in its BTB domain impairs GC formation, delays GC B cell differentiation, and disrupts antibody affinity maturation [22]. To determine whether resveratrol inhibits BCL6 function in vivo, C57BL/6 mice were subjected to intraperitoneal immunization with NP-CGG to induce GC formation. Two days later, we administered resveratrol at 100 mg/kg/d to the mice for 12 consecutive days. At the peak of GC response, mice were sacrificed. Spleens and serum were collected for functional analyses of BCL6. Flow cytometry analysis revealed that the percentage of GC B cells with resveratrol treatment reduced relative to the controls, dropping from 1.66% to 0.93% (Fig. 3A). TFH cells, which play a critical role in GC B cell maturation and differentiation and are themselves regulated by BCL6 [37], were also reduced in resveratrol-treated mice, with a marked decrease in TFH cell frequency (Fig. 3B). Additionally, a reduction in IgG1-positive cells suggested that resveratrol affects class-switch recombination (Fig. 3C). ELISA analysis of serum from treated mice demonstrated a notable reduction in NP-specific high-affinity IgG1 (NP_5_ IgG1) levels, along with decreased total IgG1 (NP_23_ IgG1) levels (Fig. 3D). These results indicate that the effects of resveratrol are consistent with the phenotype observed in BCL6-BTB mutant mice, demonstrating that resveratrol can inhibit BCL6 biological function in vivo and weaken GC formation in mice.

### *Resveratrol inhibits the proliferation of GC-derived NHL *in vitro

**Fig. 4:**
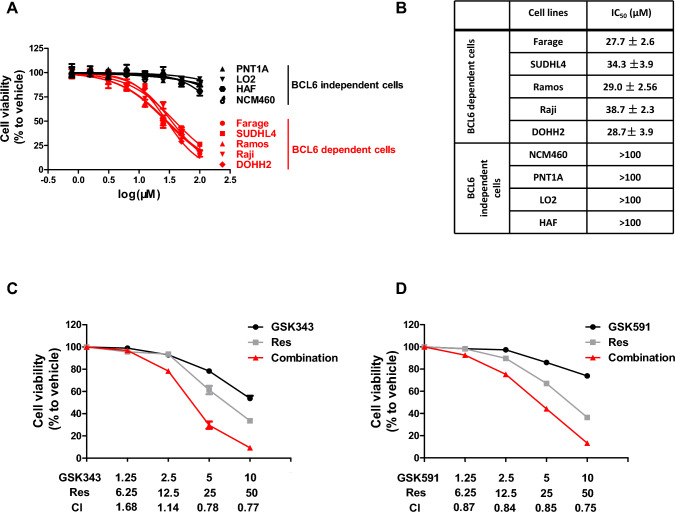
Resveratrol inhibits GC-derived NHL cells growth in vitro. **A** Anti-proliferative effects of resveratrol on GC-derived NHL cell lines and normal cell lines after 72 h of treatment. **B** IC_50_ values of resveratrol in lymphoma cell lines and normal cell lines after 72 h of treatment. **C**–**D** Resveratrol synergized with EZH2 inhibitor GSK343 or PRMT5 inhibitor GSK591 to inhibit SUDHL4 cells growth. The combination indexes (CIs) are shown at the indicated concentrations

Evaluating the proliferative capacity of cells is a fundamental method for assessing the efficacy of anti-cancer agents. To investigate the effects of resveratrol on the proliferation of GC-derived NHL, we conducted MTS assays using a panel of BCL6-dependent lymphoma cell lines, including DLBCL (SUDHL4 and Farage), BL (Ramos and Raji), and FL (DOHH2). As shown in Fig. 4A–B, resveratrol exhibited significant cytotoxicity in all five cell lines after 72 h, with IC_50_ values ranging from 25 to 40 µM. To evaluate the selectivity of resveratrol, we tested its effect on BCL6-independent normal cell lines, including human epidermal fibroblasts (HAF), normal human hepatocytes (LO2), normal colon epithelial cells (NCM460), and normal prostate epithelial cells (PNT1A). At equivalent concentrations, resveratrol showed significantly lower cytotoxicity toward these normal cell lines, with IC_50_ values exceeding 100 µM (Fig. 4A–B). These findings indicate that resveratrol selectively targets lymphoma cells with high BCL6 expression, suggesting its anti-proliferative effects are mediated through BCL6 inhibition.

Given the high heterogeneity of lymphomas, combination therapies are often employed to achieve optimal therapeutic efficacy [20]. BCL6 has been reported to co-regulate GC formation and lymphoma cell survival with EZH2 [38] and PRMT5 [39], respectively. Therefore, we tested whether combining resveratrol with the EZH2 inhibitor GSK343 or the PRMT5 inhibitor GSK591 would have a synergistic effect in SUDHL4 cells. As shown in Fig. 4C–D, the combination therapy groups demonstrated higher inhibition rates of cell proliferation than the individual drug groups. The combination index (CI) values were less than 1, indicating a synergistic effect when resveratrol was combined with GSK343 (Fig. 4C) or GSK591(Fig. 4D). Concisely, these results illustrate that resveratrol can inhibit GC-derived NHL cells growth in vitro.

### *Resveratrol inhibits GC-derived NHL growth *in vivo

**Fig. 5:**
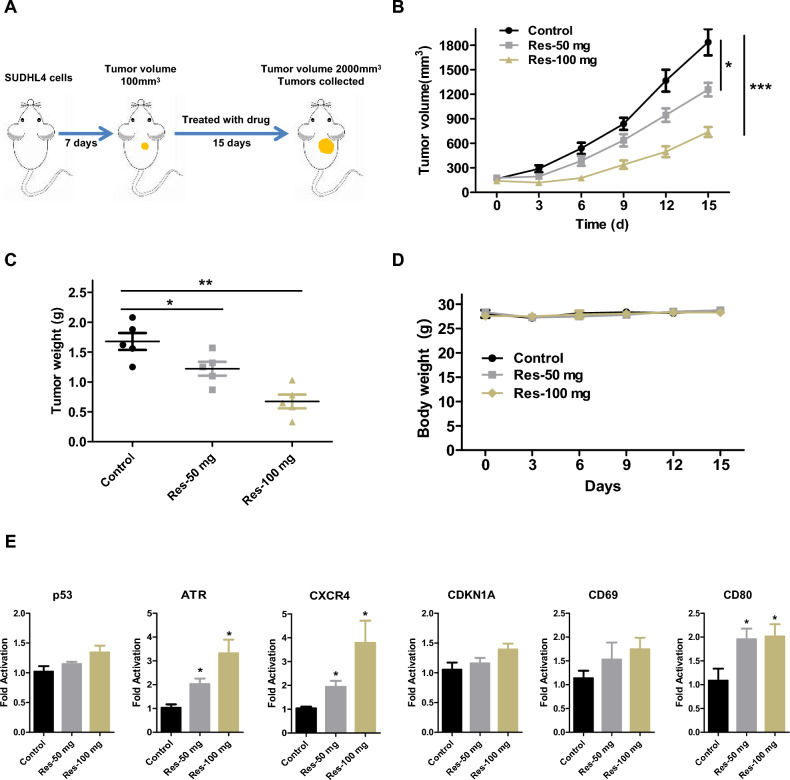
Resveratrol suppresses BCL6-driven lymphoma growth in vivo. **A** SUDHL4 xenograft mouse model. **B** Antitumor effects of resveratrol on SUDHL4 xenograft mouse model. Tumor volumes evaluated once every 3 days for a total of 15 days. **C** Tumor burden was weighed. **D** The body weight of the mice was measured every 3 days. **E** mRNA expression of the BCL6 target genes p53, ATR, CXCR4, CDKN1A, CD69 and CD80 from tumors was measured by RT-qPCR assays. (*, *P* < 0.05; **, *P* < 0.01; ***, *P* < 0.001 versus control)

Building upon the observed anti-proliferative effects of resveratrol in *vitro*, we next investigated its therapeutic potential in vivo using a subcutaneous tumor model. DLBCL cells SUDHL4 were injected subcutaneously into immunodeficient mice to establish tumors (Fig. 5A). Based on preliminary experiments, mice were randomized into three groups: control (vehicle only), low-dose resveratrol (50 mg/kg/day), and high-dose resveratrol (100 mg/kg/day). Mice were administered resveratrol intraperitoneally for 15 consecutive days, during which tumor volumes and body weights were recorded every three days. At the conclusion of the study, mice were euthanized, and the tumors were excised and weighed. The results showed that resveratrol inhibited lymphoma growth in a manner proportional to the dosage (Fig. 5B). The final tumor weights corroborated these findings, with the high-dose resveratrol group displaying the most substantial reduction relative to the control group (Fig. 5C). During the treatment period, all mice maintained good physical condition, and there was no significant weight loss (Fig. 5D). Additionally, we analyzed the expression of BCL6 downstream genes within the tumors of each group. As shown in Fig. 5E, resveratrol upregulated the expression of BCL6 downstream genes, consistent with the in vitro results (Fig. 2). In conclusion, resveratrol effectively inhibits lymphoma growth in vivo.

## Discussion

BCL6 dysregulation is a direct driver of GC-derived NHL [20, 40]. Throughout GC reaction, BCL6 represses the expression of genes associated with DNA damage and cell cycle checkpoints, enabling the conditions necessary for affinity maturation of immunoglobulins. This repression, combined with the occurrence of somatic hypermutation, increases the risk of malignant transformation in GC B cells. Chromosomal translocations and point mutations that result in uncontrolled BCL6 expression place GC B cells in a state of high proliferation, lack of differentiation, and resistance to apoptosis. Once malignant transformation occurs, this dysregulation drives GC-derived NHL [5, 41]. BCL6 overexpression is a hallmark of GC-derived NHL, making it a key therapeutic target [15]. Research indicates that targeting BCL6-BTB domain is an effective strategy for treating DLBCL, BL and FL [15, 19]. In recent years, multiple research institutions and pharmaceutical companies have been focused on developing BCL6 inhibitors, leading to the emergence of several new BCL6 inhibitors [26–28, 42–44]. However, the identification of natural inhibitors remains underexplored, and clinical research in this area remains undeveloped, highlighting the significant challenges in developing effective BCL6 inhibitors.

By constructing a HTRF high throughput screening model, we screened a series of traditional Chinese medicine monomers and identified resveratrol as an effective BCL6 inhibitor. Our studies revealed that resveratrol directly binds to the BCL6 BTB domain, thereby suppressing BCL6 function both i*n vitro* and in vivo. BCL6 functions by repressing a network of genes involved in DNA damage response (p53 and ATR), cell cycle regulation (CDKN1A), B cell activation (CD69 and CD80), and plasma cell differentiation (PRDM1). BCL6 suppresses the transcription of p53, leading to inhibition of apoptosis and disruption of DNA damage repair mechanisms. This suppression provides a survival advantage to lymphoma cells, even under genotoxic stress [33]. CDKN1A, a pivotal regulator of the cell cycle, is directly repressed by BCL6 to enable unchecked cellular proliferation, thereby driving the rapid expansion of lymphoma cells [18]. Also, BCL6 represses PRDM1, a critical factor of plasma cell differentiation, thereby maintaining B cells in an undifferentiated, proliferative state that contributes to lymphoma progression [45, 46]. Resveratrol inhibits BCL6 transcription function and upregulats the expression of BCL6 target genes both in vitro (Fig. 2) and in vivo (Fig. 5E). The upregulatory effect of resveratrol is more pronounced in vitro than in vivo, which can be attributed to several factors. Firstly, pharmacokinetics and bioavailability play a role. In vivo, resveratrol exhibits low bioavailability due to its rapid metabolism and systemic clearance [47, 48]. Secondly, cellular microenvironment influences the outcomes. In vivo studies occur within a complex microenvironment that includes interactions with extracellular matrix components, immune cells, and other signaling molecules. These interactions can modulate resveratrol’s effects on gene expression [49]. Thirdly, it is impacted by systemic factors. In vivo, systemic regulatory factors such as endocrine signals, immune responses, and multi-organ interactions influence the activity of resveratrol [50]. In vitro studies bypass these limitations and complexities, leading to more direct and pronounced gene regulation. Besides, resveratrol disrupts BCL6 biological functions in vivo*.* In NP-CGG immunization models, resveratrol disrupted GC formation by reducing GC B and TFH cell populations, impairing class-switch recombination and high-affinity antibody production (Fig. 3). This phenocopies the loss-of-function effects observed in BCL6-deficient mice, affirming its on-target activity.

Resveratrol as a BCL6 inhibitor, strongly inhibits the proliferation of lymphoma cells (Fig. 4A-B). Additionally, resveratrol exhibited significant antitumor effects in lymphoma xenograft models (Fig. 5B–C). Given the intrinsic heterogeneity of lymphomas, combination therapies are often required to maximize therapeutic efficacy. Currently, several strategies involving the combination of BCL6 inhibitors with several agents, including PRMT5 inhibitors [39], EZH2 inhibitors [38], STAT3 inhibitors [51] and chemotherapy drug doxorubicin [27], have been explored for the treatment of lymphomas. We have validated the synergistic effects of combining resveratrol with EZH2 and PRMT5 inhibitors (Fig. 4C–D). Future studies will explore additional combination drug strategies to further improve therapeutic outcomes.

Interestingly, BCL6 overexpression extends beyond lymphomas, being implicated in breast cancer [34], ovarian cancer [[35], glioma [36], chronic myeloid leukemia [52], non-small cell lung cancer [53] and so on. Preliminary findings manifest that resveratrol reactivates BCL6 downstream targets in solid tumor cell lines, suggesting its potential utility in broader oncological contexts (Supplementary Fig. 3). Extending these studies to additional cancer models will elucidate resveratrol’s role in modulating BCL6 activity across diverse malignancies, potentially expanding its clinical applications.

## Conclusion

In summary, by constructing a HTRF screening model, we identified resveratrol as a BCL6 inhibitor from traditional Chinese medicine monomers. Resveratrol effectively disrupts BCL6 biological functions both in vitro and in vivo by targeting the BCL6 BTB domain, significantly inhibiting the proliferation of GC-derived NHL cells. This highlights resveratrol’s potential for clinical research and underscores its broader applicability to GC-derived NHL.

## Supplementary Information

Below is the link to the electronic supplementary material.

Supplementary file1 (DOCX 260 KB)
