# Supplementary material for: Resveratrol as a BCL6 natural inhibitor suppresses germinal center derived Non-Hodgkin lymphoma cells growth
Source: J Nat Med. 2025 Jan 15;79(2):399–411. doi: 10.1007/s11418-024-01873-4 (PMC11880072; doi:10.1007/s11418-024-01873-4)
Supplement: Supplementary file 1 — Supplementary file1 (DOCX 260 KB) [file 11418_2024_1873_MOESM1_ESM.docx]

**Supplementary Figures**

**Figure S1. (A) Chemical structure of FX1. (B) FX1 and resveratrol attenuated BCL6^BTB^-mediated transcriptional repression in luciferase reporter assays. (*, P < 0.05; ***, P < 0.001 versus control).**

**Figure S2. Resveratrol binds to BCL6-BTB mutants L25A and Y58A.**

**Figure S3. Resveratrol induces derepression of BCL6 target genes p53, ATR, CXCR4, CDKN1A, CD69 and CD80 in cell lines MDA-MB-231, ES-2 and U87.** **(*, P < 0.05; **, P < 0.01; ***, P < 0.001 versus control).**
